# Perfluorinated Ionomer Dispersion Preparation: Autoclaving vs. High-Pressure Homogenizing

**DOI:** 10.3390/membranes16030083

**Published:** 2026-02-26

**Authors:** Sofia M. Morozova, Nataliia V. Talagaeva, Nadezhda N. Dremova, Ulyana M. Zavorotnaya, Andrey S. Starikov, Nikita A. Emelianov, Evgeny A. Sanginov, Alexander M. Korsunsky, Alexey V. Levchenko, Alexey V. Vinyukov

**Affiliations:** 1 Moscow Center for Advanced Studies, Kulakova Str. 20, Moscow 123592, Russia; 2 Federal Research Center for Problems of Chemical Physics and Medical Chemistry of the Russian Academy of Sciences, Academician Semenov Ave., 1, Chernogolovka 142432, Russia; talagaevanv@mail.ru (N.V.T.); dremova@icp.ac.ru (N.N.D.); um.zavorotnaia@misis.ru (U.M.Z.); andreistarikov1994@mail.ru (A.S.S.); nikita_emelyanov@bk.ru (N.A.E.); sanginov@icp.ac.ru (E.A.S.); 3 National University of Science and Technology MISIS, Leninskiy Prospekt, 4, Moscow 119049, Russia; 4 Center for Engineering Systems and Sciences, Bolshoi Boulevard, 30, Moscow 121205, Russia; alexander.korsunsky@structuralintegrity.eu

**Keywords:** Nafion, Aquivion, perfluorosulfonic acid dispersion, film casting, high-pressure homogenizer

## Abstract

Perfluorinated sulfonic acid ionomer (PFSAI) dispersions are widely used for fabrication of ion-conducting membranes and catalyst layers for hydrogen fuel cells. The conformation and concentration of PFSAIs affect the properties of the final product and depend on the liquid phase in dispersion. Here we present a novel method of preparing water/alcohol dispersions based on Nafion and Aquivion PFSAI by using a high-pressure homogenizer. The proposed route is faster and much safer and allows achieving higher PFSAI concentrations in comparison with the autoclave technique used for commercial dispersion preparation. The comparison of dispersion viscosity and PFSAI aggregate size was performed for both techniques and demonstrated similar values. Analysis of the morphology of membranes obtained from different dispersions by the casting method revealed differences in structure, which disappeared after annealing. These results highlight an important novel method of preparing PFSAI dispersions and the use of membrane morphology analysis for membrane quality evaluation.

## 1. Introduction

Perfluorosulfonic acid ionomers (PFSAI) are in great demand for the production of hydrogen–air fuel elements and electrolyzers [1,2,3,4,5,6]. In these devices, PFSAI is both present as a membrane separating the anode from the cathode and incorporated into the catalyst layers. The most promising and studied perfluorinated polymer for the mentioned applications is Nafion and its analogues, in which combinations of crystalline hydrophobic tetrafluoroethylene fragments and amorphous sulfonic acid-containing fragments lead to the formation of unique proton-conducting channels [2,7,8]. While the structure of Nafion dry and water-swelling membranes and analogues has been actively and extensively studied [9,10,11], the dispersion parameters of Nafion, Aquivion, and analogues in liquid media have not been thoroughly investigated [12,13]. The conformation of PFSAI in the dispersions affects the structure and morphology of the resulting ion-conducting membranes [14,15,16] and their properties, such as ionic conductivity, hydrogen permeability, and mechanics [17]. Dispersions are also used to produce catalytic layers, where the conformation of the polymer and its interaction with the particles of the catalyst are also important [18,19,20].

Existing work shows that, depending on the liquid phase, PFSAI can be in the form of a solution with different copolymer conformations, i.e., sphere and cylinder [21,22], or as dispersions with aggregates [14,23]. It has been shown that Nafion exists in the form of aggregates in an aqueous alcohol medium, as cylinders in glycerol and as true solutions, i.e., copolymer coils, in aprotic solvents of the N-methyl pyrrolidone type (NMP) [24]. However, Nafion’s conformation in various liquid media has not been reliably established for Aquivion, and despite the difference only in the length of the side chain, Aquivion exhibits greater ionic conductivity than Nafion, greater crystallinity, and a different softening temperature [25,26,27]. In addition to the nature of the ionomer and the nature of the liquid medium of the dispersion or solvent, the concentration of the ionomer is affected, which, as a result of the polyelectrolyte effect, can lead to significant conformational changes [28]. Dispersion analysis includes the scattering methods Small-Angle X-Ray Scattering [20,29], Small-Angle Neutron Scattering [30,31], Dynamic Light Scattering (DLS) [32], modelling [23,33] and analysis of rheological parameters with a focus on viscosity [34,35,36]. However, the majority of work focuses either only on dispersion or only on the final product (membrane, catalytic layer).

Dispersions from PFSAI are usually prepared by the autoclave method with [37] PFSAI heating at 80 °C and intensive mixing [12] or by using the ultrasound technique [38]. The dispersion itself can serve as a control for evaluating the quality of the copolymer obtained. PFSAIs are obtained by emulsion polymerization in the presence of gaseous tetrafluoroethylene [2,10,39]. The polymer obtained in the form of latex is precipitated, washed, stabilized, hydrolyzed, and then used for dispersion preparations [40].

For the first time, we demonstrate the possibility of obtaining a homogeneous dispersion of PFSAI using a high-pressure homogenizer (HPH). We compare commercial Nafion and Aquivion dispersions prepared by the autoclave method and dispersions prepared by the high-pressure homogenizer technique. We performed analysis of both the dispersions themselves and the morphology of the resulting films. It has been shown that the membranes obtained by different methods after annealing have the same morphology. Due to the high rate of dispersion production, the achievement of higher concentrations of PFSAI, and greater safety, the HPH method is a promising alternative to the autoclave method.

## 2. Materials and Methods

### 2.1. Materials

Nafion^®^ dispersion (20 wt%, water/1-propanol, DuPont, Wilmington, DE, USA), Aquivion^®^ water/alcohol dispersion (25 wt%, water, SolvaySolexis, Brussels, Belgium), HyProof^®^ (commercially available powder, Shanghai, China), 1-propanol (special purity, Chimmed, Moscow, Russia), isopropyl alcohol (special purity, Chimmed, Russia), and deionized water were used as received.

### 2.2. Redispersion of Commercial Polymers

Dispersions were generally prepared by passing a pre-prepared ionomer powder suspension through an ultra-high-pressure homogenizer SCIENTZ-207A at 100 MPa. The suspension was made in a 1/1 water-isopropyl alcohol mixture.

For measurement purposes, commercial dispersions of Nafion^®^ DE1020 and Aquivion^®^ D98-25BS were diluted to a concentration of 1% using a 1/1 mixture of water and isopropyl alcohol. For the HyProof ionomer, the initial suspension was prepared by adding commercially available powder (16% by weight) to a 1/1 water/isopropyl alcohol solvent mixture. The mixture was then shaken and subjected to ultrasound treatment for 30–45 min in an ultrasonic water bath with a power of 110 W and an operating frequency of 35 Hz. For preparation of Nafion and Aquivion high-pressure homogenizer-based dispersions, the commercial dispersions were dried, and the resulting powders were annealed for 180 min at 130 °C and 150 °C, respectively. Weighed portions of the resulting powders (2% by weight) were placed in a 1/1 water/isopropyl alcohol solvent mixture, followed by shaking and ultrasonic treatment for 30–45 min in an ultrasonic water bath with a power of 110 W and an operating frequency of 35 Hz. After passing through a homogenizer for 30 min at a pressure of 100 MPa, the resulting target dispersions were purified from mechanical impurities by filtration using a hydrophilic filter and sedimentation in a centrifugal field (20,000× *g*).

### 2.3. Dispersion Characterization

Dynamic light scattering analysis (DLS): The electrokinetic potentials (ζ-potential) and the hydrodynamic diameter of the copolymer aggregates were determined using DLS (Photocor Compact Z instrument, Photocor LCC, Moscow, Russia). Experiments were performed at 22 °C in triplicate format, and the correlation data were analyzed by the non-negative least squares method. Dispersions diluted up to 0.1–0.5 wt% were used.

Viscosity measurements: The viscosity of the samples was measured at room temperature on an SV-10 vibrating viscometer (A&D Company, Tokyo, Japan) at a vibration frequency of 30 Hz and a constant amplitude of less than 1 mm. For measurements, 10 mL samples were placed in special plastic cuvettes. The density of the sample under study was later used to calculate the value of its dynamic viscosity. The viscosity measurement procedure for each sample was performed until a constant viscosity value was set on the instrument display; if there was a change in values in a certain steady-state range for ~5 min, the boundaries of the range were recorded, and the average value for this range was used for calculations. To calculate the dynamic viscosity, the average value was divided by the density of the corresponding sample.

Scanning electron microscopy (SEM): The morphology was studied using a Zeiss SUPRA 25 scanning autoemission electron microscope (Carl Zeiss NTS GmbH, Oberkochen, Germany) at an accelerating voltage of 4.5 kV. The samples were obtained by applying pre-diluted wt% ionomer dispersions on a silicon substrate followed by drying at room temperature. The samples were obtained by applying 10 μL of pre-diluted 0.2 wt% ionomer dispersions on a silicon substrate on area of about 1 cm^2^ (the estimated thickness was about 0.1 μm) followed by drying at room temperature. If necessary, individual samples were annealed. The samples were annealed at a temperature above the glass transition temperature, T_g_. Due to the different length of the side chain, the T_g_ values of Nafion and Aquivion differ and are equal to 105 and 130 °C, respectively [41]. Before being placed in the microscope chamber, the samples were coated with a layer of carbon.

Atomic force microscopy (AFM): The scans were performed in the semi-contact AFM mode on an Ntegra Prima atomic force microscope using an FMG-01/Pt cantilever (NT-MDT LCC, Moscow, Russia) with a typical probe radius of 35 nm and a resonant frequency of 66 kHz. The scans were performed on areas of 4 × 4 microns (512 × 512 pixels).

## 3. Results

### 3.1. PFSAI Dispersion Preparation

**Figure 1:**
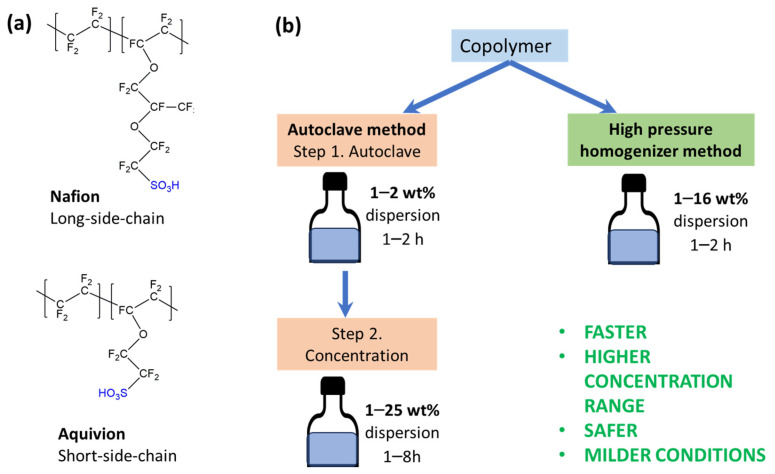
(**a**) Chemical structure of perfluorinated ionomers; (**b**) scheme of dispersion analysis.

While perfluorinated ionomers are widely available in commercial products, their dispersions are less common commercially. Here we use commercially available PFSAI dispersions based on long-side-chain (LSC) copolymer Nafion (Figure 1a top, Table 1, sample #1) and short-side-chain (SCC) copolymer Aquivion (Figure 1a down, Table 1, sample #2).

Commercial dispersions, namely Nafion-comA and Aquivion-comA, were produced by an autoclave method that includes at least two steps: heating 1–2% PFSAI dispersion in an autoclave for 1–2 h and subsequent concentration by removing the liquid medium (Figure 1b) [37,42,43].

We fabricated PFSAI dispersions by the high-pressure homogenizer one-step method (Figure 1b) from a commercial copolymer with Nafion structure (HyProof, Table 1, sample #3) and from Nafion (Table 1, sample #4) and Aquivion (Table 1, sample #5) copolymers where copolymer powders were obtained from commercial dispersions.

The autoclave method, also called the solvothermal cell method, is widely used for the preparation of dispersions due to the achievement of higher homogenization of PFSAI compared to normal-pressure heating and ultrasound [43]. However, this method requires significant time and allows obtaining only dilute (about 1% by weight) dispersions, whereas higher concentrations can be achieved via evaporation of the solvent. Also, for autoclave methods, an ionomer is often used in the form of a sodium salt, rather than in the form of an acid, which requires additional steps [44].

Here we present a new method for the preparation of PFSAI dispersion involving the use of a high-pressure homogenizer. The conducted experiments on dispersion by the developed method made it possible to obtain transparent and homogeneous dispersions of perfluorinated ionomers. The primary mechanism of operation of a high-pressure homogenizer is based on a combination of hydrodynamic and cavitation effects: the hydrodynamic effect occurs when a mixture passes through a narrow channel, where it experiences a sharp reduction in cross-section and, as a result, an increase in velocity and pressure [45]. After exiting the narrow channel, the channel expands, leading to a sharp drop in pressure and the formation of cavitation bubbles. Within the cavitation effect, when the bubbles implode, high energy is released in the form of shock waves, which exert intense mechanical force on the mixture particles, causing their fragmentation and improving homogeneity [45,46]. The combination of these two effects allows a high-pressure homogenizer to achieve high degrees of dispersion and emulsification, unattainable using traditional methods. In addition, HPH technology turned out to be 10 times faster than autoclave technology, since (i) it allows one to obtain dispersions of up to 16 wt% immediately (without concentration as in the autoclave method; see Table 1, sample HyProof-HPH), (ii) it is safer due to the small volumes and the connection of the system to the atmosphere and (iii) it does not require conversion of the polymer to the sodium form. It is worth noting that the autoclave method requires working with a closed system under pressure (~3–4 MPa for water/alcohol dispersions) and high temperatures up to 100–200 °C [44], which requires strict safety measures. Another advantage of the HPH method is scalability. Autoclave technique scale is related to autoclave size, where the HPH dispersion is passed through a HPH (thin tube) the required number of times, so a small amount of substance is involved at a certain time, but the method is flow-through.

Thus, HPH technology allows us to obtain dispersions in a shorter time and under milder conditions (room temperature, 100 MPa). The specifics of the method are presented in Table 2.

It is important to note that the concentration range of the dispersions prepared in this way largely depends on the nature of the polymer, in particular on its ion exchange capacity (equivalent mass), molecular weight and phase composition.

### 3.2. PFSAI Dispersion Characterization

**Figure 2:**
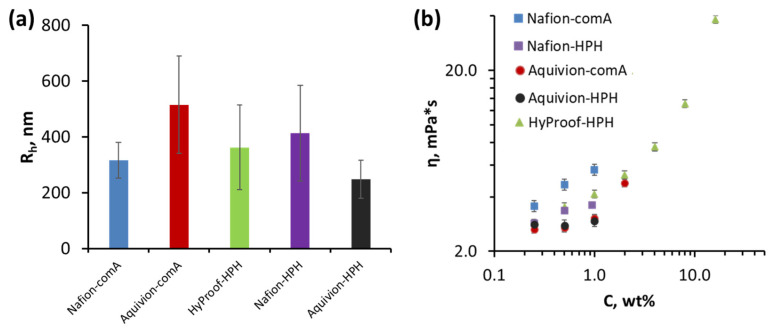
(**a**) Hydrodynamic radius of ionomer’s aggregates in dispersions measured by dynamic light scattering; (**b**) dynamic viscosity of ionomer dispersions at different concentrations.

To investigate the influence of the dispersion preparation method on the hydrodynamic radius of PFSAI, we analyzed the dispersions using dynamic light scattering (Figure 2a).

The hydrodynamic radius of particles in Nafion dispersions was 316 ± 64 nm for Nafion-comA, 412 ± 171 nm for Nafion-HPH and 362 ± 152 nm for Nafion-HyProof. This, taking into account the error, indicates similarity in the hydrodynamic radii of particles prepared by different Nafion dispersion methods. It is worth noting that the obtained data are in good agreement with the results of the work [47] and indicate the existence of ionomer molecules in the form of aggregates. For Aquivion dispersions, the results were opposite: using a homogenizer demonstrated a twofold decrease in the hydrodynamic radius of aggregate particles (248 ± 69 nm) compared to the autoclave technique (514 ± 179 nm). That is, in the case of a short-chain ionomer, the HPH method would allow for even more efficient dispersion. This behaviour of Nafion and Aquivion may be due to the different molecular weights and phase compositions of the polymers. Furthermore, it should be noted that the hydrodynamic radius is calculated based on the assumption of a spherical shape of the analyzed sample and the diffusion coefficient in the liquid, as a result of which the actual particle size may not correspond to the hydrodynamic radius. Such changes can be explained by the influence of the nature of the liquid phase. Commercial dispersions, Nafion-comA and Aquivion-comA, are aqueous, while dispersions from the homogenizer are aqueous/alcoholic. Although the ratio of alcohol and water in the water/alcohol mixture affects the conformation of the ionomer, i.e., size of aggregates [14,23], this is not as strong as a complete change in the nature of the solvent, for example, to aprotic dipolar analogues, which led to the formation of cylinders and systems close to solutions rather than dispersions [24]. In water/alcohol mixtures, agglomeration is supposed to occur through hydrophobic backbone aggregation; ionic side chains surround the surface of formed structures (primary agglomeration, particles ~1 μm). Then primary aggregation particles aggregate again through hydrophilic ionic side chains (secondary aggregations, particles ~10 μm). The size of non-aggregated particles is about 0.1 μm, but they are typical for polar solvents [47,48]. Thus, the difference in particle sizes in commercial dispersions (Nafion and Aquivion) may be related to the following. Considering that Aquivion ionomer has a higher crystallinity, it can be expected to have a more significant contribution from the hydrophobic backbone aggregation, leading to the formation of larger particles, compared to Nafion ionomers [49]. The aggregated form of the ionomer in the dispersion is also confirmed by the zeta potential data (Table S1, Supporting Information). All dispersions have a zeta potential in the range of −23 to −42 mV, which corresponds to a stable colloidal system. However, overall, it can be concluded that the HPH method provides a sufficiently high degree of homogenization compared to the autoclave method.

A comparison of the dynamic viscosity measured at 22 °C (Figure 2b) and density (Tables S2 and S3, Supporting Information) for all the obtained dispersions in the concentration range of 0.25–1.0% revealed no significant differences depending on the preparation method. However, a slight increase in dynamic viscosity of 7–8% was noted for Aquivion dispersions prepared by the HPH method. At the same time, the viscosity of Nafion and HyProof dispersions prepared by the HPH method (No. 3, HyProof-HPH, and No. 4, Nafion-HPH) was virtually identical, while dispersion No. 1, Nafion-comm, demonstrated a 20% higher viscosity. This difference in the behaviour of similar sulfonic cation exchange polymers with short and long side chains may, we believe, be due to differences in the phase composition of the polymers and their molecular weights.

Thus, based on the analysis of the dispersion properties of the obtained dispersions, it can be concluded that there is no significant difference between autoclaving and the high-pressure method (HPH).

### 3.3. Morphology of Films Prepared from Dispersions by Casting

**Figure 3:**
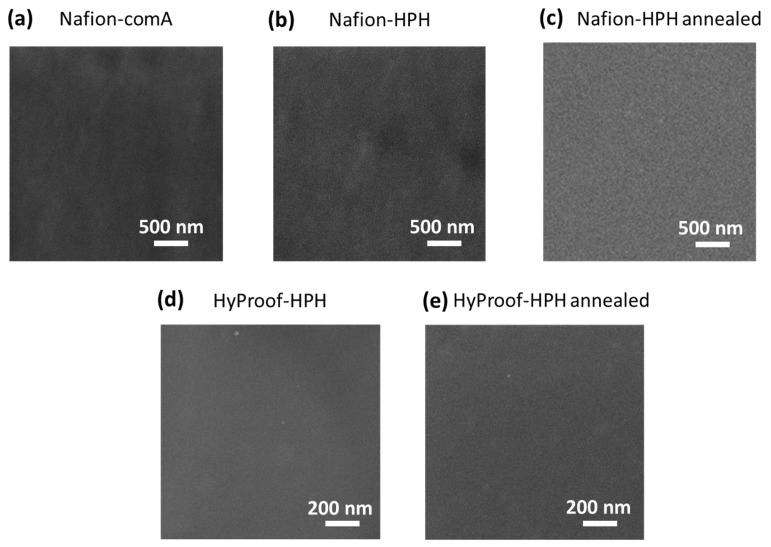
Scanning electron microscopy images for (**a**) Nafion-comA, (**b**) Nafion-HPH and (**c**) Nafion-HPH annealed at 130 °C for 3 h; (**d**) HyProof-HPH, and (**e**) HyProof-HPH annealed.

Next, we have prepared films from dispersions by the casting method and analyzed their microstructure by scanning electron microscopy to estimate film morphology and homogeneity. Figure 3a–c demonstrate the ionomer dispersions for Nafion-based ionomers.

**Figure 4:**
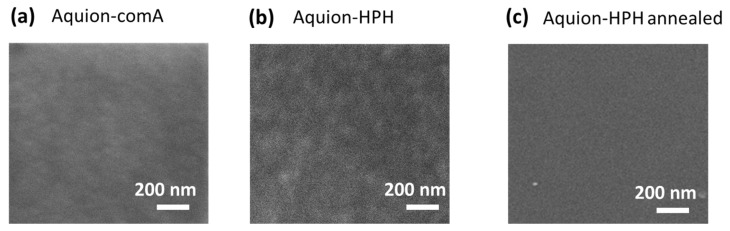
Scanning electron microscopy images for (**a**) Aquivion-comA, (**b**) Aquivion-HPH and (**c**) Aquivion-HPH annealed at 130 °C for 3 h.

Films from the commercial Nafion dispersion (Figure 3a) obtained by the autoclave method and films from Nafion-HPH and HyProof-HPH dispersions obtained on a high-pressure homogenizer (Figure 3b,d) have a similar homogeneous structure. All show slight irregularities on the film surface; however, the films prepared from HPH dispersions are characterized by a higher degree of irregularity. Annealing the Nafion-HPH film above the glass temperature of the copolymer allows for a homogeneous film (Figure 3c), similar in structure to film obtained from a commercial dispersion (Figure 3a). In the case of the ionomer with a low equivalent weight, HyProof, obtained by the HPH method, no visible changes in the film morphology are observed after annealing (Figure 3d,e). This also indicates the influence of the nature of the ionomer on the conditions for obtaining dispersions. The result was similar for films based on Aquivion dispersions (Figure 4). Both films obtained from the autoclave commercial dispersion and using the HPH technique showed a well-defined heterogeneous structure, but the HPH method resulted in a slightly more pronounced heterogeneity (Figure 4). The observed inhomogeneities disappeared with subsequent annealing.

**Figure 5:**
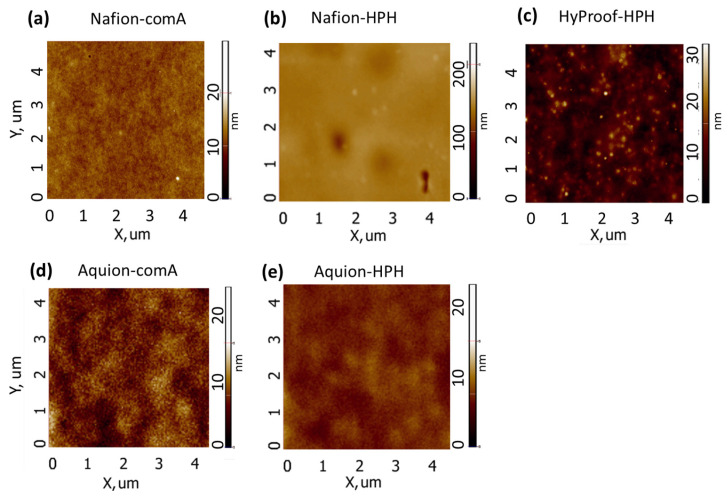
Atomic force microscopy images for (**a**) Nafion-comA, (**b**) Nafion-HPH, (**c**) Nafion-HPH, (**d**) Aquivion-comA, and (**e**) Aquivion-HPH. All films were annealed at 130 °C for 3 h.

The morphology of the obtained films after annealing was also studied by atomic force microscopy (AFM). Images of AFM films obtained from both commercial dispersions (Nafion-comA, Aquivion-comA) and those prepared using HPH (Nafion-HPH, Aquivion-HPH) have a similar morphology (Figure 5).

The HyProof-HPH sample differs somewhat in morphology from other samples; it has local aggregates of several tens of nm (Figure 5c), although this may be due to the nature of the ionomer.

Thus, it can be concluded that, despite potential challenges with polymer redispersion using the HPH method, film annealing allows for good results, i.e., homogeneous films. Consequently, the method for producing dispersions based on perfluorinated sulfonic cation exchange polymers using a high-pressure homogenizer is promising compared to the autoclave method due to its speed and the ability to achieve high ionomer concentrations in the dispersions. 

## 4. Conclusions

In this work we demonstrate a novel method of PFSAI dispersion fabrication using a high-pressure homogenizer. A comparison is made between commercially available PFSAI-based dispersions and those prepared utilizing the autoclave method and the high-pressure homogenizer (HPH) technique with both Nafion and Aquivion. It is shown that the viscosity of the dispersions differs by no more than 10% for both methods for both short-side-chain Aquivion and long-side-chain Nafion copolymers. An analysis of the size of aggregates in dispersions revealed various trends: for Nafion, the size of aggregates was almost independent of the method of obtaining the dispersion, while for Aquivion, the high-pressure homogenizer made it possible to obtain aggregates twice as small as the autoclave method. The analysis of the morphology of thin films by the SEM method revealed structural heterogeneities for films obtained from HPH dispersions in comparison with homogeneous films from autoclave-type dispersions. However, it was shown that annealing above the softening temperature of the polymer (in this work, annealing was performed at 130 °C for Nafion and 150 °C for Aquivion) made it possible to achieve homogeneous films. Thus, HPH method is a more safe method which allows for a reduction in the time of dispersion preparation by one order of magnitude compared to the autoclave method, as well as achieving higher concentrations (subsequent concentration is used for the autoclave method).

This work presents an important step for producing PFSAI dispersions and understanding solvent/ionomer interactions. Dispersion-level interactions persist across all length scales, affect film formation and properties, and are maintained upon thermal annealing. More importantly, one can contemplate engineering dispersions and inks for specific PFSAI properties, thus enabling higher-performing devices such as fuel cells and electrolyzers.

## Figures and Tables

**Table 1:** Description of used samples.

#	Sample Name	Ionomer Type	Concentration, wt%	Liquid Media	Fabrication Method
1	Nafion-comA	Nafion	10	water	Commercial dispersion ^1^. Autoclave preparation and concentration
2	Aquivion-comA	Aquivion	25	water	Commercial dispersion ^2^. Autoclave preparation and concentration
3	HyProof-HPH	Nafion	16	water/isopropyl alcohol mixture 1/1	Prepared by HPH from commercial copolymer
4	Nafion-HPH	Nafion	1	water/isopropyl alcohol mixture 1/1	Prepared by HPH from copolymer obtained from #1
5	Aquivion-HPH	Aquivion	1	water/isopropyl alcohol mixture 1/1	Prepared by HPH from copolymer obtained from #2

^1^ by DuPont; ^2^ by Solvay Solexis (not manufactured at this moment).

**Table 2:** Comparison of autoclave and high-pressure homogenizer techniques for dispersion preparation.

Parameter	Autoclave	High-Pressure Homogenizer
Form of ionomer	In most cases Na^+^ form (sodium salt)	H^+^ form (acid)
Stages of preparation	Transformation of ionomer to the form of sodium salt (~0.5 h) Autoclave preparation of dispersion (~2 h with cooling) Transformation of ionomer to the acid form (~0.5–1 h) Concentration by solvent evaporation (1–8 h)	HPH preparation of dispersion (~1 h) Centrifugation (~0.5 h)
Safety	Closed system with high internal pressure, precautions must be taken	It is possible to achieve a higher pressure, but due to the small volumes and the connection of the system to the atmosphere, the method is safer
Achievable pressure	3–5 MPa	100 MPa
Temperature	100–230 °C [37,43]	20–50 °C
Scalability	Depends on the reactor volume	The dispersion is passed the required number of times through a HPH (thin tube), a small amount of substance is involved at a certain time, but the method is flow-through.

## Data Availability

Data are available on request from corresponding author.

## Supplementary Materials

The following supporting information can be downloaded at: https://www.mdpi.com/article/10.3390/membranes16030083/s1, Table S1: Zeta-potential of PFSAI dispersions.; Table S2: Description of used samples; Table S3: description of commercial dispersions.

## Abbreviations

The following abbreviations are used in this manuscript:

AFM	Atomic force microscopy
PFSAI	Perfluorinated sulfonic acid ionomer
NMP	N-methyl pyrrolidone
DLS	Dynamic Light Scattering
SEM	Scanning electron microscopy
LSC	Long-side-chain
SSC	Short-side-chain
HPH	High-pressure homogenizer
