# Supplementary material for: Perfluorinated Ionomer Dispersion Preparation: Autoclaving vs. High-Pressure Homogenizing
Source: Membranes (Basel). 2026 Feb 26;16(3):83. doi: 10.3390/membranes16030083 (PMC13027717; doi:10.3390/membranes16030083)
Supplement: Supplementary file 1 [file membranes-16-00083-s001.zip › membranes-4099939-supplementary.pdf]

membranes

MDPI

Supplementary Material

Perfluorinated Ionomer Dispersion Preparation: Autoclaving vs. High-Pressure Homogenizing

Sofia M. Morozova [1,*], Nataliia V. Talagaeva [2], Nadezhda N. Dremova [2], Ulyana M. Zavorotnaya [2,3], Andrey S. Starikov [2], Nikita A. Emelianov [2], Evgeny A. Sanginov [2], Alexander M. Korsunsky [4], Alexey V. Levchenko [2,*] and Alexey V. Vinyukov [2,*]

1 Moscow Center for Advanced Studies, Kulakova Str. 20, 123592 Moscow, Russia
2 Federal Research Center for Problems of Chemical Physics and Medical Chemistry of the Russian Academy of Sciences, Academician Semenov Ave., 1, 142432 Chernogolovka, Russia; talagaevanv@mail.ru (N.V.T.); dremova@icp.ac.ru (N.N.D.); um.zavorotnaia@misis.ru (U.M.Z.); andreistarikov1994@mail.ru (A.S.S.); nikita_emelyanov@bk.ru (N.A.E.); sanginov@icp.ac.ru (E.A.S.)
3 National University of Science and Technology MISIS, Leninskiy Prospekt, 4, 119049 Moscow, Russia
4 Center for Engineering Systems and Sciences, Bolshoi Boulevard, 30, 121205 Moscow, Russia; alexander.korsunsky@structuralintegrity.eu
* Correspondence: sofiionova@yandex.ru (S.M.M.); a.levchenko@icp.ac.ru (A.V.L.); vinyukovav@icp.ac.ru (A.V.V.); Tel.: +7-(985)9108502 (S.M.M.)

S1. Zeta-potential of dispersions

Table S1 presents data of zeta-potential of prepared dispersions. All dispersions have a zeta potential in the range of -23 to -42 mV, which corresponds to a stable colloidal system.

Table S1. Zeta-potential of PFSAI dispersions.

#	Samplename	Zeta potential, mV
1	Nafion-comA	−27 ± 2
2	Aquivion-comA	−35 ± 3
3	HyProof-HPH	−37 ± 3
4	Nafion-HPH	−23 ± 2
5	Aquivion-HPH	−42 ± 4

S2. Density and viscosity of dispersions

Table S2 summarize density and viscosity for prepared dispersions and viscosity for commercial samples based on datasheet.

Table S2. Description of used samples.

#	Samplename	Density, g/cm^3 (1 wt%)	Viscosity ^{a}, mPa·s	Viscosity ^{b}, mPa·s
1	Nafion-comA	0.928	5.7	2-10 (10 wt%)
2	Aquivion-comA	0.917	3.1	< 25 (20 wt%)
3	HyProof-HPH	0.913	4.1	Not applicable
4	Nafion-HPH	0.937	3.6	Not applicable
5	Aquivion-HPH	0.951	2.9	Not applicable

^{a} measured; ^{b} by datasheet from Dupont (for Nafion-comA, sample D1020 [1]) and Solvay Solesix (for Aquivion-com [2]).

 https://doi.org/10.3390/membranes16030083

S3. Density and viscosity of commercial dispersions

Table S3 summarize selected parameters for commercial dispersions based on respective datasheets, for Nafion-comA [1] and Aquivion-comA [2].

Table S3. Description of used samples.

#	**Parameter**	Nafion-comA[a]	**Aquivion-comA [b]**
1	Polymer content, wt%	10–12	25
2	Water content, wt%	87–90	75
3	Volatile organic compound content, wt%	<1	Not applicable
4	Specific gravity	1.05–1.07	Not applicable
5	Total acid capacity, meq/g	1.03–1.12	0.98–1.06
6	Viscosity, mPa·s (25 °C)	2–10	<25z
7	Density, g/cm^3 at 20°C	Not applicable	1.15
8	Equivalent weight, g/eq	Not applicable	940–1020

[a] based on data from datasheet sample D1020 [1]; [b] by datasheet from Dupont (for Nafion_comA, Appendix A) and Solvay Solesix (for Aquivion-comA, Appendix B).

References

[1] https://www.fuelcellearth.com/wp-content/uploads/converted_files/pdf/D1021.pdf?srsltid=AfmBOorD0TsmnQE1U4X_uupuB-dIkxKucl4JHFVCV8E7ysqgXI9GRPnmK date of request 17.02.2026

[2] https://www.fuelcellstore.com/spec-sheets/solvay-aquivion-d98-25bs-dispersion.pdf date of request 17.02.2026
